# Effects of *Xeroderma pigmentosum group C* polymorphism on the likelihood of prostate cancer

**DOI:** 10.1002/jcla.23403

**Published:** 2020-06-02

**Authors:** Yidan Yan, Jianmin Xu, Bin Xu, Qiaxian Wen, Jing Zhou, Lifeng Zhang, Li Zuo, Guoqiang Lv, Yunfeng Shi

**Affiliations:** ^1^ Department of Oncology Affiliated Hospital of Jiangnan University Wuxi China; ^2^ Department of Gastrointestinal Surgery Affiliated Hospital of Jiangnan University Wuxi China; ^3^ Department of Urology The Affiliated Changzhou No. 2 People's Hospital of Nanjing Medical University Changzhou China; ^4^ Department of Urology Wujin Hospital Affiliated Jiangsu University Changzhou China

**Keywords:** analysis, prostate cancer, variant, *XPC*

## Abstract

**Background:**

Numerous studies have assessed the association between *xeroderma pigmentosum complementation group C (XPC)* polymorphisms and susceptibility of prostate cancer (PCa); however, the findings remain inconsistent.

**Methods:**

We performed an updated analysis utilizing data from electronic databases to obtain a more accurate estimation of the relationship between *XPC* rs2228001 A/C polymorphism and PCa risk. We further used in silico tools to investigate this correlation.

**Results:**

Totally, 5,305 PCa cases and 6,499 control subjects were evaluated. When all studies pooled together, we detected no positive result (recessive genetic model: OR = 1.14, 95% CI = 0.93‐1.40, *P*_heterogeneity_ = 0.001, *P* = .212); nevertheless, the *XPC* rs2228001 A/C variant was associated with PCa risk in Asian descendants in the subgroup analysis (OR = 1.21, 95% CI = 1.01‐1.43, *P*_heterogeneity_ = 0.008, *P* = .034). In silico tools showed that more than 20 proteins can participate in the protein crosstalk with XPC. The expression of *XPC* was down‐regulated in all Gleason scores of prostate cancer.

**Conclusions:**

The present study indicated that the *XPC* rs2228001 A/C variant may be associated with elevated PCa risk in Asian patients.

## 1 INTRODUCTION

Prostate cancer (PCa) is the most common malignant tumor among males all over the world. Previous publications reported that PCa is the second and third leading cause of male death in the United States and Europe, respectively.^1^, ^2^ In the United States, about 174 650 new PCa cases were diagnosed and 31,620 patients died from this disease in 2019 estimated by the National Cancer Institute (https://seer.cancer.gov/csr/1975_2015). In Asian descendants, the PCa incidence and mortality rates were increasing extensively in recent years.^3^, ^4^ Up to now, the specific mechanisms and exact cause of PCa are not clear.^5^ Due to the stage of this disease and the choice of patients, the prevention and treatment of PCa remains complicated.^6^ Hence, it is necessary to demonstrate the molecular mechanism and explore new targeted therapies for PCa.

Studies have shown that genetic factors may play a crucial role in the development of PCa. Down‐regulation of DNA repair is a pivotal factor in the progression of PCa.^7^ Nucleotide excision repair (NER), a main human DNA repair pathway, is one of the most significant defense mechanism against mutagenic exposure.^8^ Xeroderma pigmentosum group C (XPC) is involved in the early damage initiation of NER.^9^ The *XPC* gene located on c3p25 of homo sapiens.^10^ Mutation of *XPC* can alter the capacity of NER and lead to carcinoma of human.^11^ The substitution of A to C at position 939 is the most widely studied single nucleotide polymorphisms in XPC.^12^

Previous publications demonstrated that the *XPC* rs2228001 A/C variant may be associated with increased risk for colorectal, bladder, breast, and lung cancer.^13^, ^14^, ^15^, ^16^ Association between this *XPC* variant and PCa likelihood was previously assessed.^17^, ^18^, ^19^, ^20^ However, there are vague conclusions about the relationship between *XPC* rs2228001 A/C polymorphism and PCa susceptibility in different case‐controlled studies. Hence, a systematic analysis based on all eligible studies was conducted to further investigate the correlation between the *XPC* rs2228001 A/C polymorphism and PCa risk.^17^, ^18^, ^19^, ^20^, ^21^, ^22^, ^23^, ^24^, ^25^, ^26^, ^27^, ^28^

## 2 METHODS AND MATERIALS

### 2.1 Search strategy

We performed a comprehensive literature search on electronic databases including Web of Science, Cochrane Library, Google Scholar, PubMed, EMBASE, and Chinese Biomedical Database to retrieve all publications on the *XPC* rs2228001 A/C polymorphism and PCa susceptibility. The search terms were as follows: “XPC OR xeroderma pigmentosum group C,” “polymorphism OR single nucleotide polymorphism OR mutation OR variant,” and “carcinoma OR tumor OR adenocarcinoma OR cancer.” The last search was updated on April 10, 2020. We further screened the supplementary material of accept articles to maximize the search.

### 2.2 Study selection and inclusion criteria

Two investigators independently searched the studies according to inclusion criteria. The inclusion criteria were as follows: (a) evaluating the association between the *XPC* rs2228001 A/C polymorphism and PCa risk; (b) including available genotype frequencies to calculate odds ratio; and (c) using a case‐control design.

### 2.3 Exclusion criteria

The exclusion criteria were as follows: (a) Data of control were not available; (b) with no available genotype frequency for the *XPC* rs2228001 A/C polymorphism; (c) review articles; and (d) duplication with overlapping data from the same authors.

### 2.4 Data extraction

For every included study, the following information was extracted: name of author, year of publication, control source, ethnicity, genotyping method, PSA level (ng/mL), age range, sample size of case and control, genotyping data of the *XPC* rs2228001 A/C variant, and *P*‐value of Hardy‐Weinberg equilibrium (HWE) for case and control. Any disagreement should be addressed by discussion with a third investigator to achieve a final decision.

### 2.5 Methods for quantitative synthesis

Odds ratio and 95% confidence interval were adopted to evaluate the correlation between the *XPC* rs2228001 A/C polymorphism and PCa risk. Four genetic models were employed in the current analysis: allelic comparison (C‐allele vs A‐allele), heterozygous contrast (CA vs AA), dominant genetic model (CC + CA vs AA), and recessive model (CC vs CA + AA). *P*‐value of heterogeneity was calculated by the *Q* test. If *P*‐value for *Q* tests more than .005, a fixed‐effect model (Mantel‐Haenszel method) was applied. On the other hand, a random‐effect model (DerSimonian‐Laird method) was conducted. Publication bias was checked by Egger's test and Begg's plot. Moreover, sensitivity analysis was applied to examine the impact of each study on the combined OR *P*‐value of HWE was detected by chi‐square test. *P*‐value more than 0.05 indicates an HWE balance. The subgroup analysis included ethnic types and source of control. The above analyses were conducted utilizing Stata software (Stata Corporation, Lakeway, TX, v11.0).

### 2.6 Expression of *XPC* utilizing in silico analysis

Online gene expression database was applied to further investigate the expression of *XPC* in PCa tissues and control (http://ualcan.path.uab.edu/). A total of 497 PCa participants and 52 controls were included for investigating the *XPC* expression. The Cancer Genome Atlas (TCGA) samples were also employed to assess the effect of *XPC* expression in PCa based on patients’ Gleason score. Furthermore, we adopt the online String server (http://string‐db.org/) to explore the protein‐protein correlation regarding *XPC*. Protein Variation Effect Analyzer (PROVEAN, v1.1) was employed to evaluate the mutation of the *XPC* rs2228001 A/C variant in human (http://provean.jcvi.org/seq_submit.php). Gene‐gene interaction of *XPC* was also investigated by TCGA samples (http://ualcan.path.uab.edu/analysis.html).

## 3 RESULTS

### 3.1 Study Characteristics

As described in Table 1, a total of 12 publications based on 13 case‐controlled studies evaluating the *XPC* rs2228001 A/C polymorphism were retrieved in our analysis. Finally, 5305 PCa patients and 6,499 control subjects were included in the present study. Moreover, we checked the minor allele frequencies (MAF) of *XPC* reported in the genome aggregation database (gnomAD, https://www.ncbi.nlm.nih.gov/snp/rs2228001#frequency_tab): for global population, 0.367; Europeans, 0.391; Asians, 0.356; Americans, 0.294; Africans, 0.270; Ashkenazi Jewish, 0.472; and others, 0.396. Therefore, in the subgroup analysis by race, a total of seven studies were based on Asian populations, three studies were based on European populations, two analyzed African descendants, and the remaining was on Arabians. In the subgroup analysis by the source of control, there were six hospital‐based studies, and the rest seven studies focused on population‐based controls. The classic genotyping method, PCR‐restriction fragment length polymorphism (RFLP), was conducted in seven of the studies.

**Table 1:** Basic information of included studies for *XPC* rs2228001 A/C variant and PCa risk

Author	Year	Source	Ethnicity	Method	PSA level (ng/mL)		Age (years)		Case				Control				Case	Control
					Case	Control	Case	Control	CC	CA	AA	*P*_HWE_	CC	CA	AA	*P*_HWE_		
Perloy	2018	PB	European	iPLEX assay	NA	NA	61.7 ± 4.1	61.2 ± 4.2	130	477	392	0.420	298	815	607	0.390	999	1720
Said	2018	HB	African	PCR‐RFLP	mean 111.41	2.225 ± 1.5	71.8 ± 11.3	69.0 ± 8.51	16	55	39	0.632	26	158	82	<0.001	110	266
Wang	2017	PB	Asian	RT‐PCR	NA	NA	NA	NA	131	459	414	0.831	125	495	435	0.379	1004	1055
Kahnamouei	2016	HB	Asian	PCR‐RFLP	9.95 (7.05‐16.5)	2.80 (1.9‐9.1)	mean 61.7	mean 69.2	47	59	47	0.005	62	88	55	0.044	153	205
Zhang	2014	HB	Asian	MassARRAY	NA	NA	66.7 ± 8.2	67.3 ± 7.5	33	38	158	<0.001	31	37	170	<0.001	229	238
Mirecka	2014	PB	European	RT‐PCR	mean 12.0	mean < 4.0	mean 68.3	mean 64.6	98	290	214	0.988	122	384	265	0.380	602	771
Sorour	2013	HB	Arabian	PCR‐RFLP	mean 48.0	mean < 4.0	65.4 ± 8.7	NA	9	25	16	0.888	5	27	18	0.263	50	50
Mandal	2012	PB	Asian	PCR‐RFLP	221 ± 57.4	2.3 ± 0.8	62.6 ± 8.9	59.1 ± 10.4	28	71	93	0.022	16	94	114	0.570	192	224
Mittal	2012	PB	Asian	PCR‐RFLP	221 ± 57.4	2.3 ± 0.8	66.0 ± 5.46	64.7 ± 5.71	28	73	94	0.031	19	104	127	0.727	195	250
Liu	2012	HB	Asian	PCR‐RFLP	161.45 ± 464.15	0.81 ± 0.90	70.7 ± 8.4	70.4 ± 10.0	31	85	86	0.196	19	100	102	0.426	202	221
Agalliu	2010	PB	European	AB	NA	NA	NA	NA	205	595	457	0.628	190	600	461	0.819	1257	1251
Agalliu	2010	PB	African	AB	NA	NA	NA	NA	16	61	70	0.623	9	38	36	0.827	147	83
Hirata	2007	HB	Asian	PCR‐RFLP	NA	NA	68 ± 5.0	67 ± 15	10	78	77	0.090	23	70	72	0.372	165	165

Abbreviations: AB, Applied Biosystems; HB, hospital‐based; HWE, Hardy‐Weinberg equilibrium; NA, not available; PB, population‐based; PCR‐RFLP, polymerase chain reaction and restrictive fragment length polymorphism; PSA, prostate‐specific antigen; RT, real‐time.

### 3.2 Quantitative synthesis

**Figure 1:**
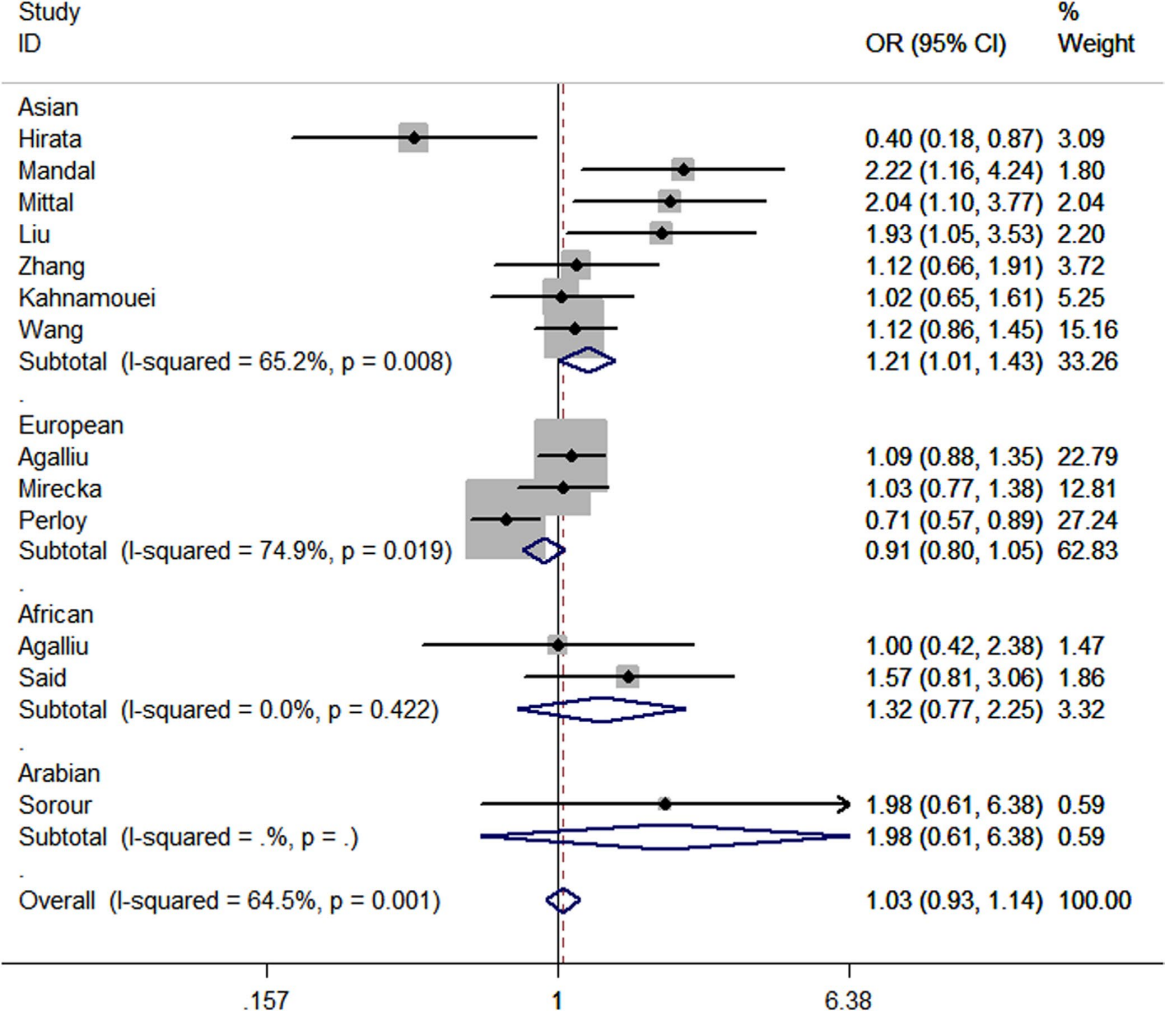
Forest plot of cancer risk correlated with *XPC* rs2228001 A/C variant (CC vs CA + AA) in stratified analysis by race

**Figure 2:**
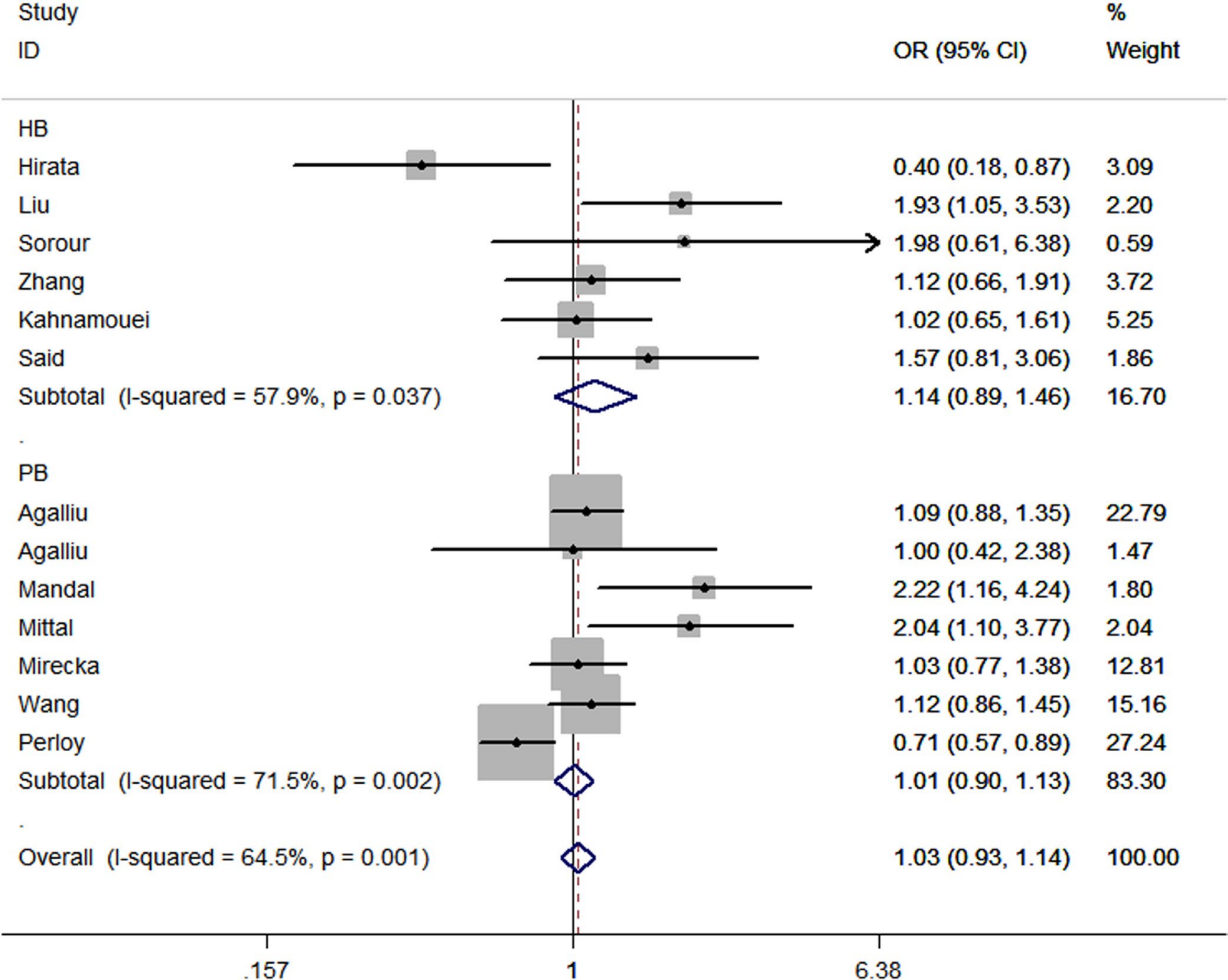
Forest plot of CC vs CA + AA model of *XPC* rs2228001 A/C polymorphism in the subgroup analysis by source of control

When all the studies pooled together (Table 2), no positive result was observed (C‐allele vs A‐allele, OR = 0.99, 95% CI = 0.94 ‐ 1.04, *P*_heterogeneity_ = 0.058, *P* = .708; heterozygous contrast, OR = 0.95, 95% CI = 0.87 ‐ 1.03, *P‐*value for heterogeneity = 0.994, *P* = .194; dominant genetic model, OR = 0.96, 95% CI = 0.89 ‐ 1.04, *P‐*value for heterogeneity = 0.837, *P* = .343; recessive model, OR = 1.14, 95% CI = 0.93 ‐ 1.40, *P*_heterogeneity_ = 0.001, *P* = .212). In a stratified analysis by ethnicity, a considerable increased risk was observed in Asian populations (OR = 1.21, 95% CI = 1.01 ‐ 1.43, *P*_heterogeneity_ = 0.008, *P* = .034, *I* ^2^ = 65.2, Figure 1). However, we observed no obvious association between *XPC* rs2228001 A/C variant and PCa risk in European populations (allelic contrast: OR = 0.94, 95% CI = 0.88 ‐ 1.01, *P*_heterogeneity_ = 0.033, *P* = .107; heterozygous contrast: OR = 0.95, 95% CI = 0.85 ‐ 1.06, *P‐*value for heterogeneity = 0.720, *P* = .333; dominant model: OR = 0.93, 95% CI = 0.84 ‐ 1.03, *P*_heterogeneity_ = 0.260, *P* = .179; recessive model: OR = 0.91, 95% CI = 0.80 ‐ 1.05, *P*_heterogeneity_ = 0.019, *P* = .199). Additionally, no positive association was identified in African individuals (allelic contrast: OR = 0.97, 95% CI = 0.75 ‐ 1.24, *P*_heterogeneity_ = 0.709, *P* = .785; heterozygous comparison: OR = 0.77, 95% CI = 0.53 ‐ 1.12, *P‐*value for heterogeneity = 0.754, *P* = .169; dominant model: OR = 0.82, 95% CI = 0.58 ‐ 1.18, *P*_heterogeneit_ = 0.918, *P* = .287; CC vs CA + AA: OR = 1.32, 95% CI = 0.77 ‐ 2.25 *P*_heterogeneity_ = 0.422, *P* = .306). Moreover, in the subgroup analysis according to the source of control, no positive association of this *XPC* polymorphism was found in population‐based studies (recessive model: OR = 1.01, 95% CI = 0.90 ‐ 1.13, *P*_heterogeneity_ = 0.002, *P* = .879, Figure 2). No positive correlation was detected in hospital‐based studies (allelic contrast: OR = 1.03, 95% CI = 0.90 ‐ 1.18, *P*_heterogeneity_ = 0.305, *P* = .660; heterozygous comparison: OR = 0.94, 95% CI = 0.77 ‐ 1.15, *P‐*value for heterogeneity = 0.814, *P* = .544; CC + CA vs AA: OR = 0.98, 95% CI = 0.82 ‐ 1.18, *P*_heterogeneity_ = 0.740, *P* = .858; CC vs CA + AA: OR = 1.14, 95% CI = 0.89 ‐ 1.46, *P*_heterogeneity_ = 0.037, *P* = .289).

**Table 2:** Stratified analysis of XPC rs2228001 A/C polymorphism on PCa risk

Variables	N ^a^	Case/Control	OR(95% CI) *P*_heterogeneity_ *P*	OR(95% CI) *P*_heterogeneity_ *P*	OR(95% CI) *P*_heterogeneity_ *P*	OR(95% CI) *P*_heterogeneity_ *P*
			C‐allele vs A‐allele	CA vs AA	CC + CA vs AA	CC vs CA + AA
Total	13	5305/6499	0.99 (0.94‐1.04) 0.058 0.708	0.95 (0.87‐1.03) 0.994 0.194	0.96 (0.89‐1.04) 0.837 0.343	1.14 (0.93‐1.40) 0.001 0.212
Ethnicity						
Asian	7	2140/2358	1.06 (0.97‐1.16) 0.208 0.177	0.97 (0.85‐1.10) 0.981 0.643	1.02 (0.91‐1.15) 0.896 0.710	1.21 (1.01‐1.43) 0.008 0.034
European	3	2858/3742	0.94 (0.88‐1.01) 0.033 0.107	0.95 (0.85‐1.06) 0.720 0.333	0.93 (0.84‐1.03) 0.260 0.179	0.91 (0.80‐1.05) 0.019 0.199
African	2	257/349	0.97 (0.75‐1.24) 0.709 0.785	0.77 (0.53‐1.12) 0.754 0.169	0.82 (0.58‐1.18) 0.918 0.287	1.32 (0.77‐2.25) 0.422 0.306
Arabian	1	50/50	1.28 (0.73‐2.26)‐0.387	1.04 (0.44‐2.48)‐0.926	1.20 (0.52‐2.74)‐0.673	1.98 (0.61‐6.38)‐0.255
Source of control						
HB	6	909/1145	1.03 (0.90‐1.18) 0.305 0.660	0.94 (0.77‐1.15) 0.814 0.544	0.98 (0.82‐1.18) 0.740 0.858	1.14 (0.89‐1.46) 0.037 0.289
PB	7	4396/5354	0.98 (0.93‐1.04) 0.029 0.547	0.95 (0.87‐1.04) 0.986 0.249	0.96 (0.88‐1.04) 0.608 0.338	1.01 (0.90‐1.13) 0.002 0.879

Abbreviations: HB, hospital‐based; PB, population‐based.

^a^ Number of case‐control studies.

### 3.3 Expression of *XPC* utilizing in silico analysis

**Figure 3:**
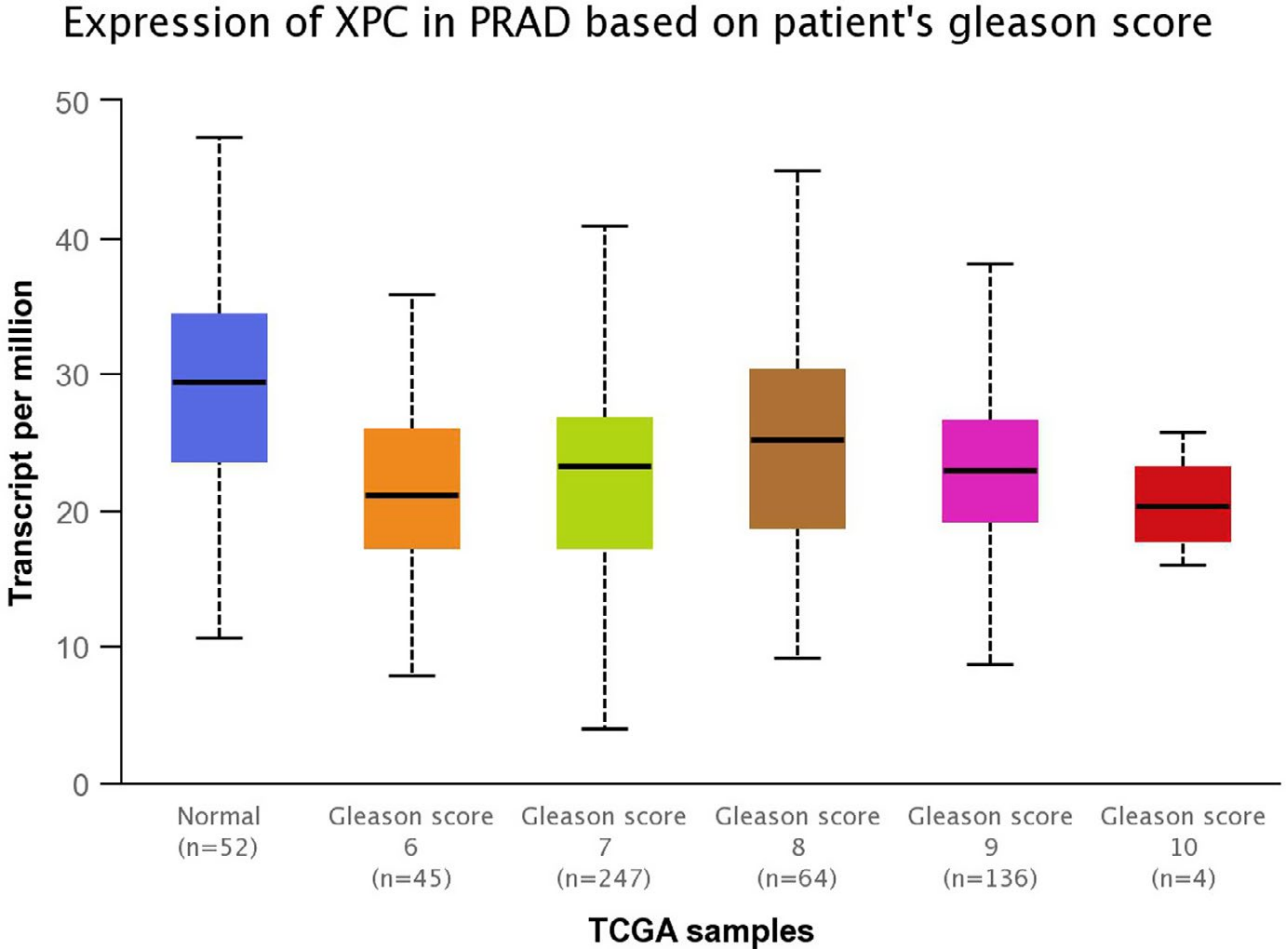
In silico analysis of *XPC* expression in PCa subjects based on patients’ Gleason score

**Figure 4:**
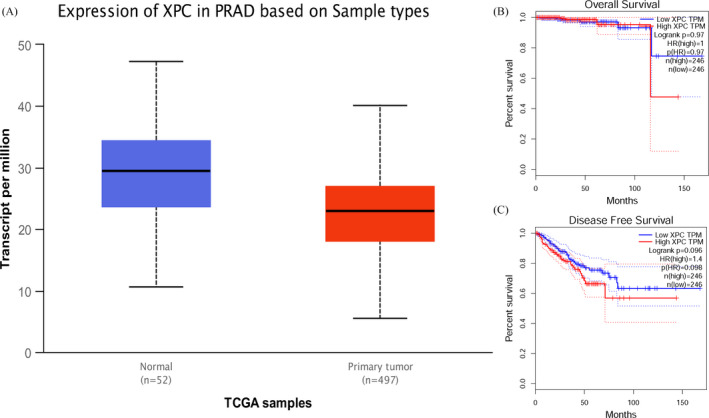
Association of the *XPC* expression in PCa based on sample types (Figure A) and the overall survival (Figure B) and disease‐free survival probability (Figure C)

**Figure 5:**
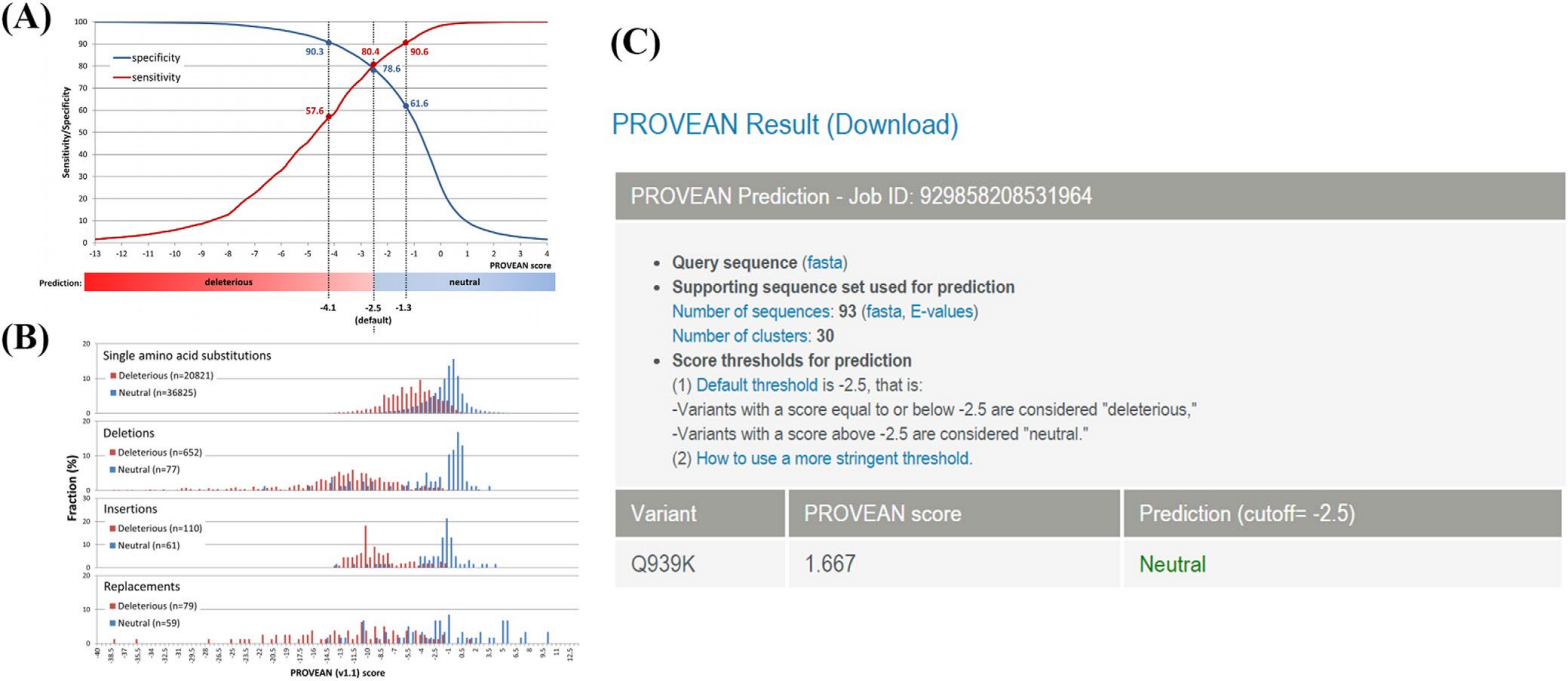
Evaluation of the *XPC* rs2228001 A/C variant by Protein Variation Effect Analyzer (PROVEAN, v1.1). Sensitivity and specificity at different PROVEAN score cutoffs are shown in Figure A (default threshold is −2.5). PROVEAN score distribution for deleterious and neutral UniProt human protein variations is shown in Figure B. The PROVEAN score of the *XPC* rs2228001 A/C variant is 1.667, which indicates that this variant is neutral (Figure C). Figure A and B is quoted from http://provean.jcvi.org/seq_submit.php

**Figure 6:**
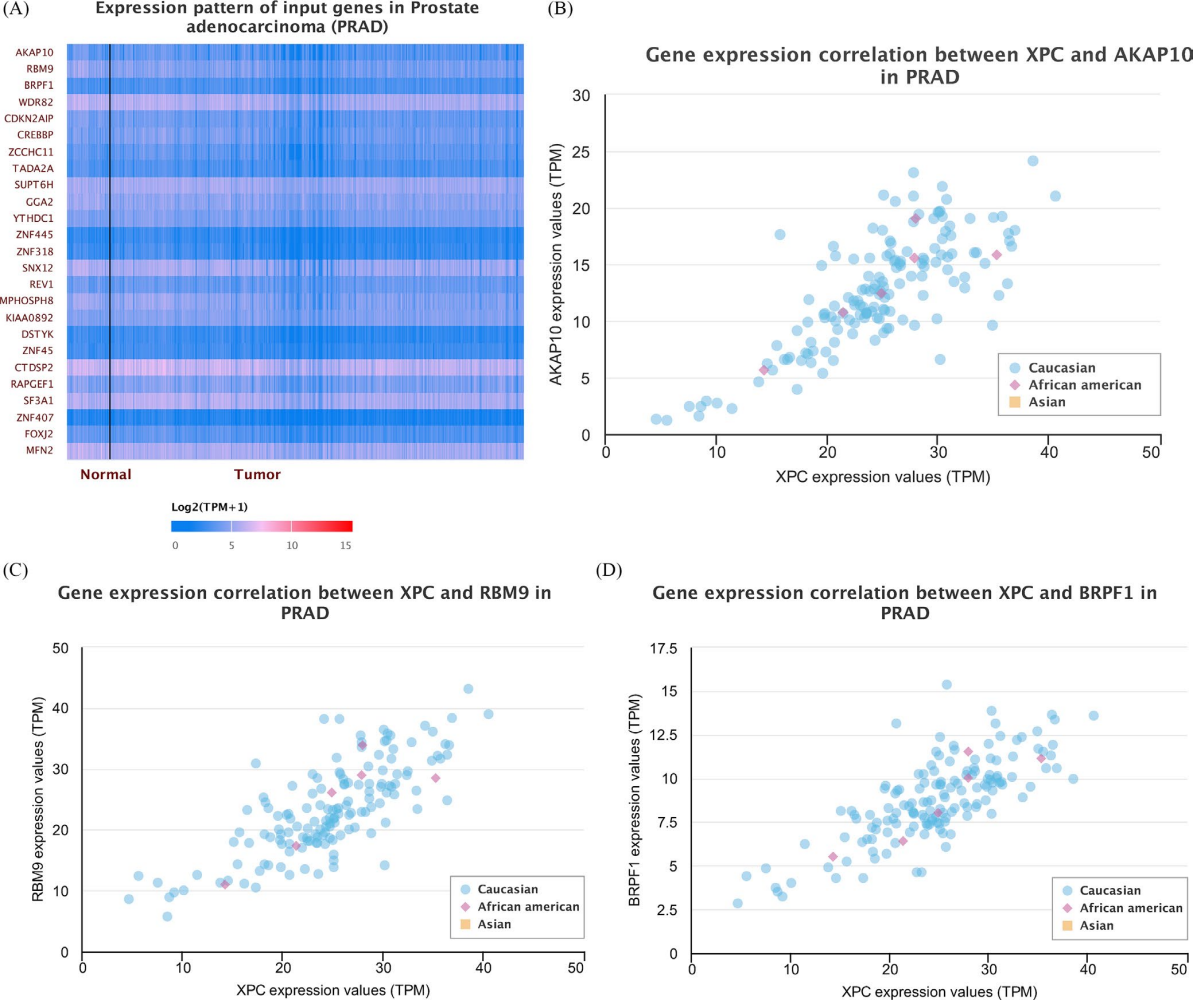
Gene‐gene crosstalk of *XPC*. As shown in Figure A, a total of 25 genes participate in the interaction of *XPC*. The *AKAP10* gene is the most related gene. There was a significant correlation between *XPC* and *AKAP10* in PCa (Figure B). Similar findings were indicated for *RBM9* (Figure C) and *BRPF1* gene (Figure D)

The in silico tool was used to evaluate the expression of *XPC* in 497 primary tumors and 52 normal tissues. *XPC* expression was lower in PCa tissues than in the control (*P* < .001, Figure 4A). The expression of *XPC* was down‐regulated in all Gleason scores of PCa. (*P* < .05, Figure 3). Furthermore, we investigated whether *XPC* expression influenced the overall survival and disease‐free survival rate in PCa cases. As shown in Figure 4B and Figure 4C, no significant correlation was observed between the high and low expression of *XPC* (*P* > .05). In order to investigate whether the rs2228001 A/C variant could have an impact on the expression of *XPC*, we adopted the Protein Variation Effect Analyzer (PROVEAN, v1.1) to predict the mutation of *XPC*. Sensitivity and specificity at different PROVEAN score cutoffs are shown in Figure 5A (default threshold is −2.5). PROVEAN score distribution for deleterious and neutral human protein variations is shown in Figure 5B. The PROVEAN score of the *XPC* rs2228001 A/C variant is 1.667, which indicates that this variant is neutral (Figure 5C). As shown in Figure 6A, at least 25 genes are involved in the interaction of *XPC*. The *AKAP10* (A‐kinase anchoring protein 10), *RBM9* (RBFOX2, RNA binding fox‐1 homolog 2), and *BRPF1* (bromodomain and PHD finger containing 1) gene are the top three related genes. Results from TCGA samples indicated a significant correlation between *XPC* and *AKAP10* in prostate cancer (Figure 6B). Similar findings were indicated for *RBM9* (Figure 6C) and *BRPF1* gene (Figure 6D). Nevertheless, there are few studies on their connections in present publications. We further used the online String server tools to explore the protein‐protein correlation regarding XPC. As described in Figure 8A, at least 20 proteins participate in the protein crosstalk with XPC. The top 10 proteins are as follows: CETN2: centrin‐2; RAD23A: UV excision repair protein RAD23 homolog A; RAD23B: UV excision repair protein RAD23 homolog B; GTF2H1: general transcription factor IIH subunit 1; XPA: DNA repair protein complementing XP‐A cells; ERCC4: DNA repair endonuclease XPF; ERCC1: DNA excision repair protein ERCC‐1; CHD1L: chromodomain‐helicase‐DNA‐binding protein 1‐like; RPA2: replication protein A 32 kDa subunit; DDB2: DNA damage‐binding protein 2 (Figure 8B).

### 3.4 Publication bias

**Figure 7:**
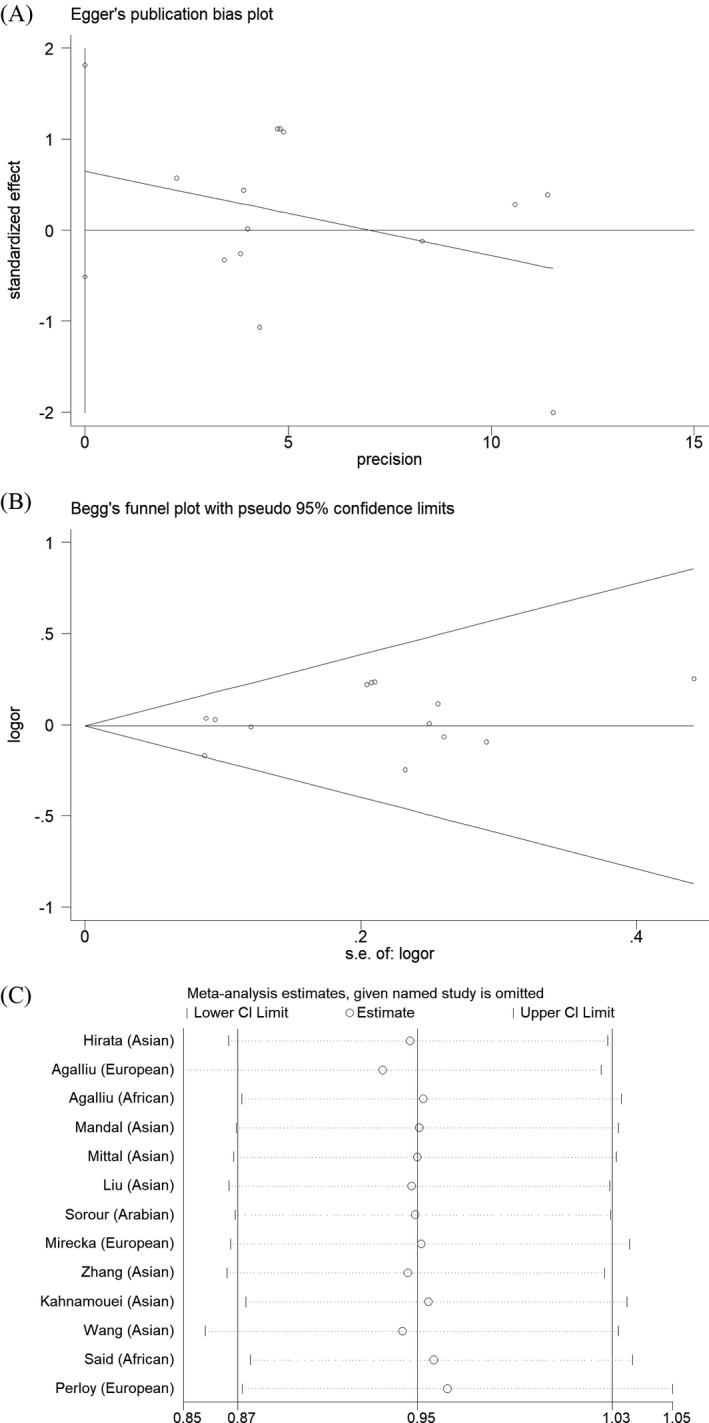
Publication bias analysis for the *XPC* rs2228001 A/C variant. No publication bias was observed from Egger's test (Figure A). The symmetry of Begg's funnel plot also indicated no evidence of publication bias (Figure B). The sensitivity analysis for the *XPC* variant is shown in Figure C. No individual study would influence the pooled OR

We conducted Egger's test and Begg's funnel plot to detect the publication bias. Moreover, sensitivity analysis was applied to examine the impact of each study on the combined OR. No publication bias for the *XPC* rs2228001 A/C variant was observed from Egger's test (Figure 7A). For C‐allele vs A‐allele: *t* = 1.30, *P* = .219; heterozygous contrast: *t* = 1.22, *P* = .246; CC + CA vs AA: *t* = 1.22, *P* = .247; CC vs CA + AA: *t* = 1.36, *P* = .202. The symmetry of the funnel plot indicated no evidence of publication bias in our analysis as described in Figure 7B. The sensitivity analysis for the *XPC* variant is shown in Figure 7C. No individual study would influence the pooled OR.

**Figure 8:**
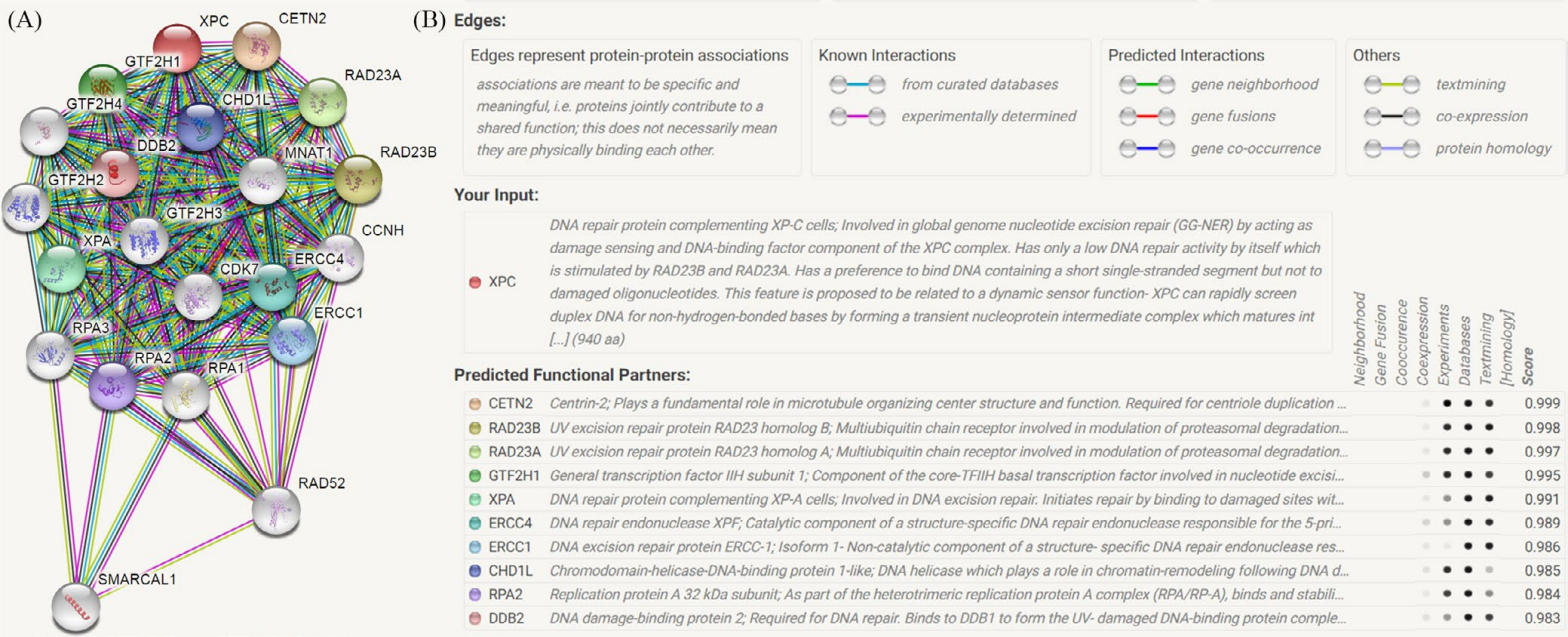
XPC correlations with other proteins determined by String server (homo sapiens). At least 20 proteins participate in the protein crosstalk with XPC (Figure A). The top 10 proteins are as follows: CETN2: centrin‐2; RAD23A: UV excision repair protein RAD23 homolog A; RAD23B: UV excision repair protein RAD23 homolog B; GTF2H1: general transcription factor IIH subunit 1; XPA: DNA repair protein complementing XP‐A cells; ERCC4: DNA repair endonuclease XPF; ERCC1: DNA excision repair protein ERCC‐1; CHD1L: chromodomain‐helicase‐DNA‐binding protein 1‐like; RPA2: replication protein A 32 kDa subunit; DDB2: DNA damage‐binding protein 2 (Figure B)

## 4 DISCUSSION

The pathogenesis of PCa remains complex. Previous research showed that genetic variants of *XPC* may be involved in down‐regulation of the DNA repair capacity (DRC).^29^, ^30^ Decreased DRC could cause genetic instability and contribute to susceptibility to PCa.^31^, ^32^ Previous case‐controlled studies were conducted to investigate whether the *XPC* rs2228001 A/C polymorphism confers the risk of PCa, but with controversial results.^17^, ^18^, ^19^, ^20^, ^21^, ^22^, ^23^ A Japanese population‐based research showed that the *XPC* rs2228001 A/C polymorphism might be a risk factor for PCa.^17^ However, another study based on Egyptian population suggested no significant difference between the *XPC* rs2228001 A/C variant and PCa susceptibility.^22^ In 2013, a meta‐analysis conducted by He *et al* indicated elevated colorectal, lung, and bladder cancer susceptibility correlated with this polymorphism.^33^ However, their conclusions cannot be confirmed by other researchers two years later.^34^ Since then, new case‐control studies have emerged. The aim of the present study was to summarize all eligible data to draw more accurate conclusions.

In this study, 5305 cases and 6,499 control subjects were finally included to evaluate the effect of the *XPC* rs2228001 A/C variant in PCa susceptibility. When all studies pooled together, no positive result was observed (recessive genetic model: OR = 1.14, 95% CI = 0.93 ‐ 1.40, *P*_heterogeneity_ = 0.001, *P* = .212). However, we found that the *XPC* rs2228001 A/C variant is associated with PCa risk in Asian populations (OR = 1.21, 95% CI = 1.01 ‐ 1.43, *P* = .034). Our finding is in line with the conclusions reported by He *et al* ^33^ Furthermore, in silico analysis was used to assess the expression of *XPC* in different grade of PCa. It showed evidence that *XPC* expression was down‐regulated in all Gleason scores of prostate cancer. We also evaluated whether the *XPC* expression influenced the overall survival probability of PCa cases; however, no positive correlation was indicated.

It is necessary to mention the limitations of the current analysis. First, the sample size of included studies in the current study was relatively small, especially for subgroup analyses. Second, some covariates such as age, tumor stage and grade, and smoking exposure should be added into stratification analysis. However, raw data of the included studies were not available to further evaluate the association between the *XPC* rs2228001 A/C polymorphism and these factors. Finally, other factors including gene‐gene and gene‐environment interactions are warranted to be considered. As shown in Figure 8, *XPC* may have the connection of twenty other proteins. Furthermore, TCGA samples have shown that more than 25 genes can participate in the connection of *XPC*. The AKAP10 gene is the most related gene. There is a significant correlation between *XPC* and AKAP10 in prostate cancer. However, there is a few research on the further mechanism of this gene, which is warranted to be evaluated in the future studies. Said *et al* found that *XPC* rs2228001 A/C variant was not correlated with PCa risk individually; however, combined analysis of rs2228001 A/C and *XPC‐PAT* variants showed that *XPC* (A/C + PAT D/D) genotypes were associated with susceptibility of PCa.^27^ Additionally, some advantages of our analysis should be considered. First, all eligible data according to the inclusion criteria were summarized to investigate the relationship between *XPC* rs2228001 A/C polymorphism and PCa risk. The statistical power of the current analysis has been strengthened considerably. Second, no evidence of publication bias was identified in both Begg's funnel plot and Egger's test, indicating that conclusions of our study were stable and trustworthy.

In conclusion, our study suggested that the *XPC* rs2228001 A/C variant might contribute to elevated PCa risk in Asian patients. The expression of *XPC* was down‐regulated in PCa with different Gleason scores. In future, more large‐scale and well‐designed studies are warranted to confirm our conclusions in more detail.

## CONFLICT OF INTEREST

The authors declare no competing interests.

## ETHICAL APPROVAL

Not applicable.

## Data Availability

All data generated and analyzed during this study are included in this published article. Please contact the author for data requests.
